# Relevance and Feasibility of a “Geriatric Delirium Pass” for Older Patients with Elective Surgeries: Findings from a Multi-Methods Study

**DOI:** 10.3390/geriatrics11010010

**Published:** 2026-01-13

**Authors:** Patrick Kutschar, Chiara Muzzana, Simon Krutter, Ingrid Ruffini, Bernhard Iglseder, Giuliano Piccoliori, Maria Flamm, Dietmar Ausserhofer

**Affiliations:** 1 Institute of Nursing Science and Practice, Center for Public Health and Healthcare Research, Paracelsus Medical University, 5020 Salzburg, Austria; simon.krutter@pmu.ac.at; 2 Claudiana Research, Claudiana-College of Health Professions, 39100 Bozen, Italy or chiara.muzzana@stud.pmu.ac.at (C.M.); dietmar.ausserhofer@claudiana.bz.it (D.A.); 3 Institute of Nursing Science and Practice, Paracelsus Medical University, 5020 Salzburg, Austria; 4 Department of Geriatrics, Hospital Meran (SABES-ASDAA), 39012 Merano, Italy; ingrid.ruffini@sabes.it; 5 Department of Geriatric Medicine, University Hospital Salzburg (SALK-Campus CDK), Paracelsus Medical University, 5020 Salzburg, Austria; b.iglseder@salk.at; 6 Institute of General Practice and Public Health, Claudiana-College of Health Professions, 39100 Bozen, Italy; giuliano.piccoliori@am-mg.claudiana.bz.it; 7 Institute of General Practice, Family Medicine and Preventive Medicine, Center for Public Health and Healthcare Research, Paracelsus Medical University, 5020 Salzburg, Austria; maria.flamm@pmu.ac.at

**Keywords:** delirium, postoperative complications, aged, risk factors, perioperative care, interdisciplinary communication, intersectoral collaboration, consensus

## Abstract

**Background/Objectives**: Postoperative Delirium (POD) is a frequent complication in older patients undergoing elective surgery. Although multicomponent interventions are effective, deficits in interdisciplinary communication and intersectoral collaboration persist. This study developed and evaluated the “Geriatric Delirium Pass (GeDePa)”, a paper-based tool to systematically document risk factors for POD across care settings. **Methods**: A multi-method design was applied, comprising (i) a structured literature review, (ii) semi-structured expert interviews, and (iii) a standardized online survey utilizing the RAND/UCLA Appropriateness Method (RAM). A total of 21 healthcare professionals (general practitioners, geriatricians, anaesthetists, surgeons, and nurses) were recruited from Salzburg, Austria, and South Tyrol, Italy (2023–2024). **Results**: Healthcare professionals confirmed the GeDePa’s practical applicability for early POD risk detection across care settings. The expert rating using the RAM Disagreement Index (DI) method deemed all 45 risk factors as sufficiently relevant and, with the exemption of two risk factors (alcohol use, intraoperative complications), feasible. A detailed analysis provided a more differentiated picture, with full consensus reached for only 18 items. Several factors with uncertain consensus (e.g., cognitive impairment and polypharmacy) were retained based on strong evidence in the literature. Others were excluded despite high ratings if they were considered redundant or impractical (e.g., detailed intraoperative complications). In total, 38 of the 45 risk factors were retained. **Conclusions**: The GeDePa is a feasible and relevant tool for structured delirium risk assessment and enhancing interdisciplinary communication between primary and hospital care. The finalized German and Italian versions are now available and will undergo further testing and implementation in clinical practice.

## 1. Introduction

Delirium is an acute disturbance of attention and cognition and is a common and clinically significant complication in older patients during hospitalization [1,2,3,4,5,6]. Postoperative delirium (POD) typically develops 24–78 h after surgery and can last from one day to several weeks [7]. In acute hospitals and geriatric departments, up to 35% of patients show delirium upon admission, and up to 64% develop delirium during their stay [3,4,6,8]. The risk of delirium increases with age and geriatric conditions [4,5,9,10,11,12]. Predisposing factors include prior delirium, dementia or cognitive impairment, depression, malnutrition, dehydration, sensory loss, frailty, sarcopenia, sleep problems, and multimorbidity. Precipitating factors include environmental stress, restraints, medications such as anticholinergics or sedatives, type and duration of surgery, urinary catheters, abnormal laboratory values, infections, hypoxia, and pain [1,2,13,14,15].

POD often remains undetected or is identified too late, resulting in preventable harm [16,17,18]. It is associated with increased mortality, prolonged hospital stays, functional decline, institutionalization, negative dementia trajectories, and higher healthcare costs [1,11,16,18].

Guidelines and several hospital-based programs, such as the Hospital Elder Life Program [19] have been implemented to mitigate these consequences. Previous research has demonstrated their efficacy in reducing both the incidence and severity of POD and delirium-related adverse outcomes [2,13,19,20,21,22]. About 30–40% of delirium cases might be prevented through multicomponent strategies [19,22,23]. Nevertheless, the significant number of patients already affected at admission to the hospital underscores that preventive strategies cannot be confined to the inpatient phase.

General practitioners (GPs) often serve as the first point of contact for older patients before elective surgery and possess comprehensive knowledge of patients’ health status, comorbidities, and baseline cognitive function. This uniquely positions primary care to enable early detection of delirium risk and intervention. However, structured pre-hospital risk assessment and systematic intersectoral communication and collaboration between primary care and hospitals remain virtually absent [21,24,25]. This constitutes a substantial gap in current delirium prevention practices and represents missed opportunities for timely intervention.

To address this gap, the “Geriatric Delirium Pass (GeDePa)” was developed as a paper-based, checklist-type tool for older patients undergoing elective surgery, allowing early identification and interprofessional documentation of delirium risk factors across care sectors. In our multi-method study, we aimed:

- to develop a preliminary GeDePa version with delirium risk factors informed by the scientific literature and clinical expert knowledge,
- to evaluate the practicability, content, layout and implementation needs through qualitative interviews with healthcare professionals (HCPs),
- to quantitatively assess the relevance and feasibility of the risk factors using a RAND/UCLA-based expert survey, and ultimately
- to integrate the findings and finalize the first ready-to-use version of the GeDePa.

## 2. Materials and Methods

### 2.1. Study Design

This study applied a multi-phase, multicentre, multi-methods design to develop and evaluate the Geriatric Delirium Pass (GeDePa). The project was conducted between January 2023 and August 2025 in Salzburg, Austria, and South Tyrol, Italy, as part of the South Tyrolean Fund for the Promotion of Scientific Research (SFPR).

The GeDePa-study consisted of four preparatory, empirical, and conceptual phases:

1. Literature screening of systematic reviews and guidelines for delirium risk factors of POD in older patients to construct a preliminary GeDePa draft,
2. qualitative expert interviews with involved health care professionals to evaluate the practicability of the GeDePa and of risk factors,
3. a quantitative online survey using RAND/UCLA Appropriateness Method to assess the relevance and feasibility of the single risk factors, and to evaluate main findings from the expert interviews,
4. a final phase focusing on integration and finalization of the GeDePa.

### 2.2. Literature Screening and Construction of the Preliminary GeDePa

**Figure 1:**
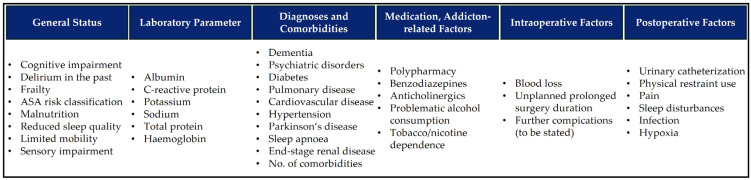
Risk factors (n = 38) for POD considered for the preliminary GeDePa.

In phase 1, a structured, pragmatic literature search was conducted in MEDLINE (via PubMed), CINAHL, and Web of Science to identify clinical guidelines, systematic, narrative, umbrella reviews, and meta-analyses on delirium risk factors and delirium risk assessment published between January 2017 and December 2022 for actuality reasons. Twenty-one publications were included (see Supplement S1 Included Literature and Risk Factors). In total, 115 risk factors were extracted, grouped, and mapped according to perioperative points of care in primary and hospital care. Our research group rated each risk factor (either exclude, or include in the GeDePa), agreement in the case of deviant ratings was established by discussion in several work meetings. Next to the core research team (PK, CM, SK, DA), the GeDePa-research group consists of scholars and clinicians with intensive academic and practical experience (BI, MF, GP, IR) with POD in geriatric patients. The prepared grouped list, the qualitatively evaluated effect strengths, and the research group’s decision of to-be-used risk factors are shown in Supplement S1. Of the 115 risk factors, we considered 38 for constructing the preliminary GeDePa (Figure 1).

Building on these findings, an initial checklist-format German version of the GeDePa was outlined as a two-page A4 sheet. It contained sections on (1) patient information (i.e., name, age, sex, primary diagnosis, planned surgery, surgery date, contact person, referring GP or clinic), (2) pre-, peri-, and postoperative risk factors for POD and healthcare profession-specific check marks, (3) filling instructions with recommendations for short versions of validated scales, and (4) an open field for comments by the responsible HCP. This preliminary, formatted GeDePa version served as the basis for subsequent empirical phases. The GeDePa was translated from German into Italian by bilingual members of the research team using a forward-translation approach, suitable for use in the qualitative interviews and quantitative online survey conducted exclusively among bilingual HCP in South Tyrol, Italy.

### 2.3. Qualitative Expert Interviews

#### 2.3.1. Participants and Recruitment

In Phase 2, semi-structured expert interviews were conducted with HCP directly involved in the perioperative care of older patients undergoing elective surgery. The professionals included GPs, geriatricians, anaesthetists, nurses, and surgeons. Inclusion criteria were at least three years of work experience and involvement in perioperative treatment and care of geriatric patients.

Recruitment followed purposive and convenience sampling strategies in Salzburg and South Tyrol. Gatekeepers, such as nursing directors and medical leads, supported recruitment. In total, 21 HCP participated in the qualitative interviews.

#### 2.3.2. Data Collection

Interviews were conducted between December 2023 and March 2024 in German or Italian. Locations as preferred by the interviewees included GP offices, hospital wards, and academic settings. The interviews lasted an average of 33 min (range: 23–46 min). The interview guide was semi-structured and covered the following thematic blocks: (1) perceived relevance of delirium in practice, (2) importance and boundaries of early risk identification, (3) practicability of GeDePa, (4) recommendations for content and design, and (5) implementation challenges. Please see Supplement S2 Interview Guide for full details. All interviews were audio-recorded, transcribed verbatim, and pseudonymized. Written informed consent, including permission for further contact via e-mail after the interviews, was obtained from all participants.

#### 2.3.3. Qualitative Data Analysis

Data were analysed using qualitative content analysis according to Mayring [26,27]. Deductive categories were based on the interview guide. Inductive categories were derived during the analysis to capture sectoral and profession-specific perspectives. Coding was carried out with MAXQDA 22 [28] by two researchers with expertise in qualitative research, followed by consensus coding and a critical discussion with additional study group members. This ensured consistency and interpretative rigor in all methodological steps in this phase.

### 2.4. Quantitative Expert Rating

#### 2.4.1. Instrument

**Figure 2:**
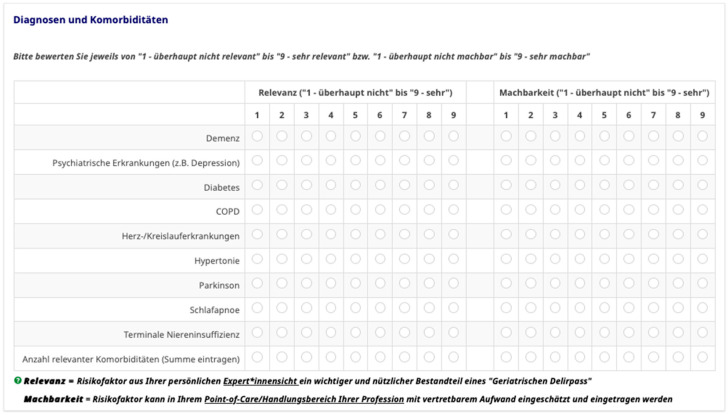
Example of the 9-point Likert-type scale ratings for relevance and feasibility for the risk factor group “Diagnoses and Comorbidities” (provided German version in LimeSurvey^TM^). * “Expertensicht” (male gender addressed) and “Expertinnensicht” (female gender addressed).

In phase 3, the list of risk factors generated in the literature screening, combined with the main findings of the qualitative expert interviews, was transferred into a standardised online survey to assess their relevance and feasibility. Implementation and analysis followed the principles of the RAND/UCLA Appropriateness Method (RAM) from Fitch et al. [29]. Next to the introductory landing pages, question instructions, and open-ended questions for further recommendations, the main part of the survey included the rating scales for the 45 risk factors, presented in matrix format and structured by the sections of the preliminary GeDePa. Risk factors were quantitatively rated for both relevance and feasibility (i.e., 90 rated items). To ensure a shared understanding among respondents, relevance was defined as the degree to which a risk factor represents an important and useful component of a “Geriatric Delirium Pass” based on the participant’s professional expertise. Feasibility was defined as the extent to which a risk factor can be assessed and entered into the GeDePa with acceptable effort at the respondent’s point of care and within their professional scope of practice. As required by the RAM framework and to allow respondents to express meaningful graduations of judgment per item, a 9-point Likert-type scale was presented for each risk factor, ranging from 1 (not relevant/not feasible) to 9 (highly relevant/highly feasible). Figure 2 depicts how the rating was implemented in the online survey, including the layout and web survey presentation style.

#### 2.4.2. Participants and Procedure

All 21 HCP from the qualitative interviews were invited to complete the online survey. Nineteen out of 21 participated and rated the risk factors for both relevance and feasibility. The survey was implemented using LimeSurvey^TM^ in May and June 2024. Participants received an invitation e-mail and had three weeks to respond. A reminder was sent after two weeks. The survey completion took approximately 15 min on average.

#### 2.4.3. Quantitative Data Analysis

Data were analysed using R Statistical Software v4.4.2 [30] by applying two different RAM procedures to measure agreement and disagreement, that is, the IPRAS disagreement method and the agreement index (AI) method. For each item, the median (Md), agreement index (AI), interpercentile range as of 70th–30th centile (IPR), and IPR adjusted for asymmetry (IPRAS) were calculated, whereby IPRAS was calculated as follows:

(1) $$\mathrm{IPRAS}\;=\;2.35+(1.5*abs(5-\frac{70th+30th\;centile}{2}))$$

where 2.35 represents the IPR required for disagreement when perfect symmetry exists, 1.5 is the correction factor for asymmetry, and *abs* indicates the absolute difference between the appropriateness score given and the panel median expressed as a positive number [29,31,32]. Next, the disagreement index (DI) was calculated as follows:

(2) $$\mathrm{DI}\;=\;\frac{IPR}{IPRAS}$$

with DI < 1 indicating absence of disagreement. The median values for each risk factor were classified as 1–3 (not relevant/not feasible), 4–6 (uncertain), or 7–9 (relevant/feasible). Items with a median of 7–9 and a DI < 1 were considered to show consensus on relevance or feasibility. Following the strict decision rules of the IPRAS method, only items rated both relevant and feasible and with measured agreement were included in the final GeDePa. To identify risk factors requiring further discussion among the expert group, we also applied the classic agreement index (AI) based on median categories (Md_cat_) [29]. The AI calculates the number of experts outside the median category to indicate agreement. According to Fitch et al., 2001, with a sample size of about 20 experts, the maximum accepted number of responses outside the median category is ≤4 [29]. Each risk factor was therefore classified as (i) consensus on both relevance and feasibility (appropriate), (ii) consensus on relevance or feasibility only (uncertain), or (iii) dissensus on relevance and feasibility (uncertain). Table 1 illustrates the classification for DI and AI methods: For frailty, both relevance (DI = 0.16) and feasibility (DI = 0.29) reached consensus based on both DI and AI, classifying the risk factor as relevant and feasible. In contrast, sleep apnoea showed higher dispersion, with many expert ratings outside the median category, resulting in DI values of 0.37 (relevance) and 0.41 (feasibility); however, classification by AI method led to uncertain agreement.

This procedure enabled the research group to identify both appropriate and potentially unnecessary risk factors for in- or exclusion in the GeDePa.

### 2.5. Finalization of the GeDePa

In phase 4, the results of the quantitative expert rating were prepared and systematically discussed within the research group. The DI was used as the primary indicator of consensus, following the RAND/UCLA conventions. AI was used as a secondary, more granular indicator to identify items with “uncertain” consensus despite DI < 1. Items classified as “uncertain” via AI were judicated by the research group. They were retained if supported by strong evidence (e.g., cognitive impairment and polypharmacy) based on our literature screening (study phase 1) and the clinical expertise of our research team members. Items were excluded from the GeDePa if they were redundant or deemed impractical (e.g., highly detailed intraoperative complications) by our clinical experts within the research team. This process resulted in the final list of risk factors, which formed the basis for the final adaptations in terms of content, layout, and specific sections of the final GeDePa.

## 3. Results

### 3.1. Sample Description

A total of 21 HCPs were interviewed, including 6 GPs, 4 geriatricians, 4 anaesthetists, 2 surgeons, and 5 nurses, with 12 participants from Salzburg and 9 from South Tyrol. On average, the participants had 18.7 years of professional experience and had worked for 7.8 years (mean M) in their current workplace. Of the 21 HCPs, 19 participated in the subsequent quantitative online survey. The survey sample comprised 10 participants from Salzburg (52.6%) and 9 from South Tyrol (47.4%). The distribution by profession was as follows: GPs (n = 5, 26.3%), geriatricians (n = 4, 21.1%), anaesthetists (n = 2, 10.5%), surgeons (n = 4, 21.1%), and nurses (n = 4, 21.1%). Although two interviewed experts from the qualitative study phase did not complete the survey, all major professional groups involved in the prevention of POD were represented.

### 3.2. Qualitative Expert Interviews

Five themes emerged during qualitative content analysis, confirming the need for a structured, accessible instrument such as the GeDePa, and directly informing the quantitative expert rating of the online survey.

- Theme 1—Measures of early identification of risk patients: All professions recognized delirium risk in older patients but emphasized different indicators. GPs pointed to dementia and substance dependence. Nurses and geriatricians emphasized age above 75 years and functional assessments. The anaesthetists noted that risk estimation rarely altered the intraoperative management. Across professions, risk detection combined formal instruments and professional judgment.
- Theme 2—Intersectoral structures and processes for delirium prevention: Interviewees reported limited integration of primary and hospital care in delirium prevention. Anaesthetists and geriatricians were seldom involved in preoperative risk assessment. Preventive processes were fragmented and varied by region. In Salzburg, the preoperative examination (‘PROP’) carried out by GPs includes relevant risk factors for POD, but similar structures were missing in South Tyrol.
- Theme 3—Information exchange in the perioperative care pathway: Gaps in communication across care sectors were described as a major challenge. Discharge letters rarely documented delirium, and feedback to GPs was inconsistent. Information was often exchanged only on an ad hoc basis by phone. In particular, the nurses highlighted the lack of systematic documentation of prior delirium episodes that would inform long-term care service providers after patient discharge.
- Theme 4—Challenges in the perioperative care pathway: Barriers included missing structures, unclear responsibilities, and limited time resources for intersectoral and interprofessional communication. Professionals criticized the insufficient preoperative assessment in primary care and the limited availability of surgeons and anaesthetists for communication after discharge.
- Theme 5—Desired processes for intersectoral care: Participants consistently wished for a shared document to record delirium risk factors and prior episodes of delirium. They envisioned a tool such as the GeDePa to accompany patients across care sectors and support prevention and management throughout perioperative care pathways. The interviewed HCP considered the length and documentation efforts of the GeDePa project to be feasible and realistic in daily practice.

Overall, the findings show that delirium prevention efforts focus mainly on risk identification within hospitals, with insufficient systematic communication and collaboration across disciplines and sectors. This study parts’ results have been published elsewhere in detail [33].

### 3.3. Quantitative Expert Rating

#### 3.3.1. Median Ratings of Relevance and Feasibility by Risk Factor Group

General Status: Delirium or confusion in the past, sensory impairment, frailty, limited mobility, reduced sleep quality, indication of malnutrition, ASA risk classification, and cognitive impairment were all rated as highly relevant (Md_Range_ 7–9) and feasible (Md_Range_ 7–9). Past delirium events, limited mobility, and cognitive impairment were rated as the most relevant and feasible risk factors.

Laboratory parameters: Albumin, C-reactive protein (CRP), potassium, sodium, total protein, and haemoglobin reached high feasibility (Md 9) and moderate-to-high relevance (Md_Range_ 7–8), with sodium and haemoglobin showing the highest relevance (Md 8).

Diagnoses and comorbidities: Neurocognitive disorders, dementia, Parkinson’s disease, and end-stage renal disease scored highest, with median ratings of 8 and 9 on the relevance dimension, while cardiovascular diseases, diabetes, hypertension, pulmonary diseases, the number of comorbidities as risk factors, and sleep apnoea received moderate ratings (Md 7). Except for the latter (Md 7), the feasibility of documenting diagnoses and comorbidities in practice was rated as very high (Md_Range_ 8–9).

Medication and addiction-related factors: Polypharmacy, benzodiazepines, and anticholinergics were rated as highly relevant (Md_Range_ 8–9). Indications for problematic alcohol consumption were rated highly relevant, while indications for tobacco/nicotine dependence rather low (Md 7). Only the indications for problematic alcohol consumption were rated moderate (Md 6) in terms of feasibility.

Intraoperative factors: Blood loss, unplanned prolonged duration of surgery, and further complications (to be filled out by the HCP) showed rather high relevance median ratings between 7.5 and 8.0, and median feasibility ratings ranging between 8.0 and 8.5.

Postoperative factors: Urinary catheterization, physical restraints, pain, infections, sleep disturbances, and hypoxia all received very high relevance ratings (Md_Range_ 8–9). Feasibility ratings ranged between 8 and 9, except for a slightly lower rating for sleep disturbance.

Further risk factors were derived from expert interviews: Type of surgery, anaesthesia technique, and bowel or bladder dysfunction were deemed relevant, with no item reaching the highest relevance (Md_Range_ 7–8). This also holds true for the evaluation of feasibility, where the lowest rating (Md 6) was given to the factor “detailed information about intraoperative complications”.

#### 3.3.2. Summary of Ratings and Indices for Expert Agreement

All 45 risk factors had median scores between 7 and 9 on the relevance scale. The disagreement index (DI) was below 1 for all items, indicating the absence of disagreement. Therefore, consensus was achieved that all risk factors were relevant for perioperative delirium prevention and management. Most risk factors also achieved median feasibility scores of 7–9. Two risk factors, alcohol use and further postoperative complications, received median scores in the range of 4–6, reflecting uncertainty, with only the latter risk factor’s DI value indicating a lack of consensus. No item was classified as irrelevant or infeasible.

**Figure 3:**
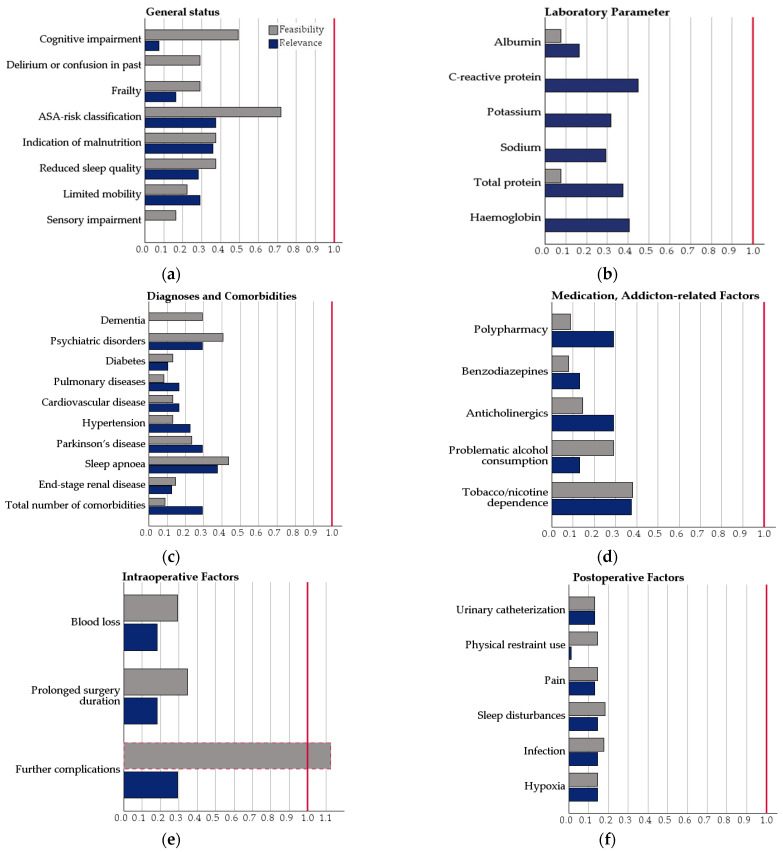

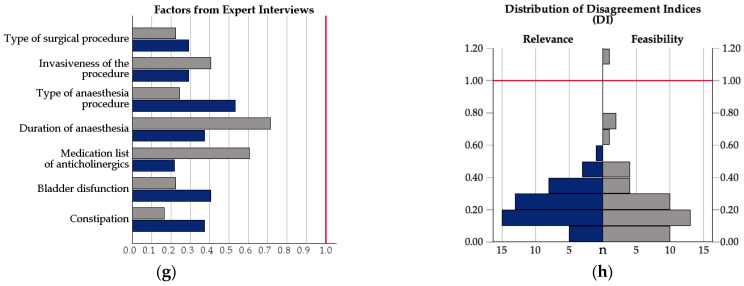
(**a**–**g**) Disagreement indices (DI) for feasibility and relevance by risk factor group. DI values near 0.0 represent the absence of disagreement. (**h**) Histogram of the total DI distribution.

Across all 45 risk factors, DI values for relevance ranged between 0.00 and 0.53 (M = 0.24, SD = 0.13), with most items clustering well below 0.30, indicating high consistency in expert ratings. For feasibility, the DI values ranged between 0.00 and 1.13 (M = 0.25, SD = 0.22). While most items fell below 0.40, a few items showed higher dispersion, with the risk factor detailed intraoperative complications showing expert disagreement (DI > 1). Figure 3a–g depicts the distribution of all DI for feasibility and relevance per group of risk factors, as well as (Figure 3h) the total DI distribution for feasibility and relevance.

To identify risk factors requiring further discussion in the research group, we applied the classic RAND/UCLA disagreement method using the agreement index (AI) based on the number of expert responses (≤4) outside the median category. Using this method, 18 risk factors were rated with a clear consensus on both relevance and feasibility (Table 2). A further 17 risk factors reached a consensus on relevance but showed uncertainty regarding feasibility, or vice versa. Ten risk factors were rated as uncertain on both dimensions.

### 3.4. Selection of Risk Factors and Finalization of the Geriatric Delirium Pass (GeDePa)

#### 3.4.1. Included and Excluded Risk Factors

The expert rating procedure based on the DI method confirmed the core set of 45 delirium risk factors as both sufficiently relevant and feasible. Enabling a more differentiated picture using the AI method, full consensus was reached for only 18 risk factors. Of those remaining risk factors, some items with uncertain consensus regarding relevance and/or feasibility were retained owing to strong evidence in the literature (e.g., cognitive impairment and polypharmacy), whereas others were excluded despite high ratings if deemed redundant or impractical (e.g., detailed intraoperative complications) by the research group. In total, 38 of the 45 risk factors were retained. Five risk factors were excluded following research group (RG) discussions, and two risk factors were excluded due to redundancy, as their content overlapped with existing items of the patient data (PD) information on the GeDePa (see Table 2).

#### 3.4.2. Adaption and Finalization of the GeDePa

Based on the findings from the expert rating, the GeDePa was revised and finalised as a two-page checklist with sections on patient data and risk factors by pre-, peri-, and postoperative phases, including a section to systematically document delirium occurrence in the postoperative phase. The German version of the GeDePa will now undergo forward-backward translation to develop the Italian version for its clinical use in South Tyrol (Italy). In the Supplement S3—Final GeDePa Version, the English version of the GeDePa (for publication uses only) can be found.

## 4. Discussion

In this multi-method study, we aimed to develop and evaluate the feasibility and relevance of the GeDePa, a paper-based checklist tool designed to optimize interdisciplinary communication and intersectoral collaboration in the prevention of delirium among older surgical patients. Based on the scientific evidence (i.e., international guidelines, systematic reviews and meta-analyses), a preliminary version of the GeDePa was developed. Semi-structured expert interviews were conducted with HCPs directly involved in the perioperative care of older patients undergoing elective surgery. Qualitative interviews with GPs, geriatricians, anaesthetists, surgeons, and nurses confirmed existing gaps in interprofessional and intersectoral communication and collaboration, as previously reported [34]. Experts across professions and sectors confirmed the overall relevance of these factors, with high feasibility ratings for most. The GeDePa was consistently described as a valuable and practical tool to overcome some of the current limitations in the detection, reporting, and prevention of POD.

The findings from the quantitative expert rating confirmed the broad agreement among HCP that delirium is a multifactorial syndrome requiring systematic risk assessment. While overall evaluations of the appropriateness of risk factors were rather positive, the RAM approach provided differentiated insights into the relevance and feasibility of the 45 risk factors identified in the scientific literature and qualitative interviews. There was a high level of agreement among experts regarding well-known risk factors, such as delirium events in the past, multimorbidity, cognitive impairment, and sensory deficits, as well as postoperative conditions, including urinary catheters, restraints, pain, and infections. These domains have consistently been reported as predictors of postoperative delirium in systematic reviews [9,10,21,22,35]. Our findings underline the robustness of these factors as well-established predictors of POD from a clinical perspective.

Several differences emerged in the assessment of the relevance and feasibility of certain factors, such as the ASA score, which is recommended as one of the key risk factors for POD by the European Society of Anaesthesiology and Intensive Care Medicine [2]. Such variations suggest that experiences differ between experts working in acute and primary care. Simultaneously, this may indicate persisting uncertainty in clinical practice and emphasise the importance of strengthening delirium knowledge among HCP across the care continuum [36,37]. Alcohol use and intraoperative complications, such as blood transfusion or fluid administration, received lower feasibility scores, despite studies reporting their predictive value [9,10]. This may reflect practical barriers in the application of such documentation tools, such as anticipated difficulties in documenting alcohol consumption in primary care and the heterogeneous nature of intraoperative complications in acute care. Standardization in clinical practice as well as translating evidence into routine practice remain challenging. Recent work relating to a nurse-led clinical pathway for delirium prevention highlights comparable barriers to consistent implementation and stresses the importance of structured approaches [38]. Hence, our decision to retain certain risk factors in the GeDePa despite lower ratings emphasizes the necessary balance between evidence and practical usability in the clinical context. Medication-related risk factors require particular attention, as they should ideally consider the total anticholinergic burden rather than the use of anticholinergic drugs [39]. Many common medications have anticholinergic properties (e.g., antidepressants, antipsychotics, and bladder control drugs) and that with even mild anticholinergic activity can have a significant cumulative effect. Thus, incorporating the total anticholinergic burden assessed with a specific scale (e.g., Anticholinergic Cognitive Burden Scale) [40] may improve future iterations of the GeDePa.

While our results show broad consistency regarding established risk factors, certain context-specific differences were observed, particularly in the primary care setting. These findings highlight the distinct contribution of the GeDePa compared with existing approaches. Tools such as the Pre-Interventional Preventive Risk Assessment (PIPRA) [41] or PROPDESC [11,42] focus on predicting delirium risk using algorithm-based or routine data, but they do not address interdisciplinary communication and collaboration across sectors to enhance preventive measures. Initiatives such as the EASE program (Elder-Friendly Approaches to the Surgical Environment) demonstrated improved outcomes with geriatric assessment and adapted care environments [43]. The Hospital Elder Life Program (HELP) has shown that multicomponent interventions can reduce the incidence of delirium by up to 40% [19]. While such approaches are typically limited to hospital settings or specialized hospital units and require considerable resources, the GeDePa fundamentally differs by offering a brief, paper-based, and intersectoral communication instrument and checklist for POD risk factors. Its novelty lies in bridging the primary care and hospital sectors by enabling GPs to document delirium risk factors before hospitalization and ensuring their transfer into in-hospital perioperative care. This addresses a major gap identified in recent work: HCP reported that risk information is rarely communicated across sectors, and that structured processes for delirium prevention are lacking across the continuum of care and along patient pathways [33]. A large-scale German survey study found that about 40% of GPs reported applying a geriatric assessment either in an unstructured or incidental way during routine consultations [44], further underscoring the lack of standardized procedures potentially linked to relevant risk factors of POD in primary care, parallel to further optimizing the existing but still in need of improvement process of standardized documentation within acute care institutions, non-speaking across the complex settings. The GeDePa directly responds to the need for a common document to systematically record and transfer delirium-relevant information and risk factors as a companion to the patient. Although the present study focused on a paper-based tool to ensure immediate usability across sectors, a digital web- or app-based version may offer advantages in terms of workflow integration, automated data transfer, and clinical decision support. Such formats and the associated hindering and facilitating factors will be explored in the upcoming implementation phase. Furthermore, our earlier qualitative study highlighted the heterogeneity in how different professional groups identify delirium risk [33]. Again, comprehensive delirium knowledge and literacy across all HCPs are needed to ensure a shared understanding of POD identification, treatment, and coordinated prevention strategies, including the involvement of caregivers and relatives [36,45,46,47]. The current study complements the current evidence by demonstrating a quantitative consensus on the core risk factors for POD while also revealing variability in the perceived feasibility of some risk factors.

### 4.1. Implications for Clinical Practice and Further Research

The findings of this study have several clinical implications. Incorporating a structured tool such as the GeDePa into routine care may facilitate early identification of older patients at-risk for POD and strengthen and standardize interdisciplinary communication between primary care and hospitals. Furthermore, the observed heterogeneity across professional groups underlines the need for targeted training and shared educational strategies to ensure a consistent level of delirium knowledge, as well as strategy for implementing the GeDePa in clinical routine. The qualitative findings showed that GPs are often not routinely involved in perioperative delirium prevention, despite their unique knowledge of patients’ treatment history. In Salzburg, the PROP examination partially fulfils this role, but no comparable structures exist in South Tyrol. The GeDePa is neither a substitute for well-known delirium screening and assessment tools (e.g., 4AT) nor for evidence-based, in-hospital risk prediction tools (e.g., PIPRA). It is a systematically developed tool to enhance interprofessional and intersectoral collaboration in the preventive identification of older patients at risk for POD after elective surgeries. From a health services perspective, we envision this practical, intersectoral application as follows: patients over 65 years of age, who are eligible for elective surgery following specialist evaluation, visit their GP to prepare for surgery (e.g., blood tests, ECG). The GP or, if involved, the outpatient clinic specialist, completes the respective part of GeDePa with the patient and their caregiver, identifying modifiable risk factors for preventive interventions. Once hospitalized, nurses, orthopaedic surgeons, and anaesthesiologists complete the profession’s respective parts of the GeDePa and assess pre-, intra- and postoperative risk factors, including delirium screening and documentation of eventual delirium events. Upon discharge, the original GeDePa is given to the patient for follow-up with the GP, and if available, a copy is also given to the outpatient nursing service provider, while a copy remains in the hospital patient records.

For further research, implementing the GeDePa is essential to assess its practical usability in real-world settings while acknowledging regional specificities and national regulations. Next, the German version will undergo full forward-backward translation and cultural adaptation, in line with established guidelines [48]. This process will be guided by best practices for clinician-reported outcome measures to ensure conceptual, semantic, and contextual equivalence between the German and Italian versions.

Moreover, outcome-based evaluations are needed to determine whether the implementation of the GeDePa can improve patient outcomes, including POD incidence and severity, length of stay, readmissions, and the quality of interprofessional and intersectoral communication. Comparative studies across different healthcare systems and settings are valuable for exploring transferability and the need for context-specific adaptations of the tool. We plan to evaluate the GeDePa in routine care, including completion time, workflow integration, and profession-specific responsibilities, and examine user preferences for layout elements, such as pictograms. Further refinement of medication-related risk factors, including the use of anticholinergic burden scores, will involve additional professional groups, such as clinical pharmacists. Digital adaptations and innovative approaches to risk prediction should be explored for integration into electronic patient records [49]. In addition to exploring the feasibility of a digital version of the GeDePa in combination with the technological readiness of HCPs at each point of care, future studies should also develop strategies to involve GPs more systematically in preoperative screening and validate whether the selected risk factor documentation across sectors predicts POD in real-world clinical settings.

### 4.2. Limitations

Several limitations must be acknowledged. First, selection bias is possible because the participating clinical experts were mainly recruited through project partners and may have had a particular interest or affinity for the topic. Second, only one round of the RAND/UCLA Appropriateness Method was applied, which may have affected the comparability of the ratings. Third, the findings are embedded within the specific health care processes of Salzburg (Austria) and South Tyrol (Italy), which may limit generalizability and transferability of the study findings to other regions or countries. Fourth, as some professional groups were underrepresented (e.g., surgeons, nursing and medical subspecialties), profession-specific bias may have been introduced in the data and results. During the initial development of GeDePa, pharmacists could have generated additional insights into medication-related risk factors. Fifth, the German–Italian GeDePa version was translated using a simplified forward-translation by bilingual team members. This was appropriate for a bilingual HCP study sample in South Tyrol, Italy, but does not meet the gold standard of independent forward–backward translation. Finally, this study focused on expert assessments rather than patient-level data, meaning that the ratings reflected subjective perceptions rather than objective measures of risk strength based on clinical data.

## 5. Conclusions

The GeDePa offers a valuable resource to strengthen interprofessional communication and collaboration between primary care and hospitals for assessing and documenting older patients’ risk factors for developing POD. HCP reported in the qualitative study phase that the GeDePa has practical applicability for early risk detection across care settings and beyond sectoral boundaries. The quantitative study phase, applying the RAND/UCLA Appropriateness Method (RAM), revealed strong agreement on the relevance and feasibility of most risk factors. Several clinically relevant but less feasible risk factors of the GeDePa were highlighted, such as certain diagnosed diseases, sleep apnoea, and alcohol or tobacco addiction. The final German and Italian versions of the GeDePa will undergo implementation and evaluation in clinical practice in South Tyrol (Italy) and Salzburg (Austria) in a consecutive study.

## Figures and Tables

**Table 1:** Example for classifying the relevance and feasibility of the risk factor “frailty” and “sleep apnoea” based on the RAND/UCLA Appropriateness Method.

Frailty	n per Category	Median (Md_cat_)	Expert Responses Outside Md_cat_ *, AI	IPR/ IPRAS	DI	Agreement (AI)
Relevance	1–3 = 2 4–6 = 0 7–9 = 17	7 (7–9)	2	1.0/6.10	0.16	Relevant
Feasibility	1–3 = 2 4–6 = 2 7–9 = 15	8 (7–9)	4	2.0/6.85	0.29	Feasible
**Sleep** **Apnoea**	**n per** **Category**	**Median** **(Md_cat_)**	Expert Responses Outside Md_cat_ *, AI	**IPR/** **IPRAS**	**DI**	**Agreement (AI)**
Relevance	1–3 = 0 4–6 = 7 7–9 = 12	7 (7–9)	7	2.0/5.35	0.37	Uncertain
Feasibility	1–3 = 3 4–6 = 6 7–9 = 10	7 (7–9)	9	2.2/5.05	0.41	Uncertain

* Median categories (Md_cat_): 1–3 (not relevant/not feasible), 4–6 (uncertain), or 7–9 (relevant/feasible); AI = Agreement Index (≤4 responses outside the median category); IPRAS = Interpercentile range adjusted for asymmetry; DI = Disagreement Index.

**Table 2:** Summary of the consensus-based rating of the relevance and feasibility of the 45 risk factors (order as presented in the GeDePa).

Risk Factors	Relevance	Feasibility	Agreement *	Decision	Reason
*General Status*					
Sensory impairment	+	-	Uncertain (|)	Inclusion	RG
2. Frailty	+	+	Agreement	Inclusion	SE
3. Limited mobility	+	+	Agreement	Inclusion	SE
4. Reduced sleep quality	-	-	Uncertain (&)	Exclusion	RG
5. Indication of malnutrition	-	-	Uncertain (&)	Inclusion	RG
6. ASA-risk classification	-	-	Uncertain (&)	Inclusion	RG
7. Delirium or confusion in the past	+	+	Agreement	Inclusion	SE
8. Cognitive impairment	+	-	Uncertain (|)	Inclusion	RG
Laboratory parameters (out of range)					
9. Albumin	-	+	Uncertain (|)	Inclusion	RG
10. C-reactive protein	-	+	Uncertain (|)	Inclusion	RG
11. Potassium	-	+	Uncertain (|)	Inclusion	RG
12. Sodium	-	+	Uncertain (|)	Inclusion	RG
13. Total protein	-	+	Uncertain (|)	Inclusion	RG
14. Haemoglobin	-	+	Uncertain (|)	Inclusion	RG
Diagnoses and comorbidities					
15. Dementia	+	-	Uncertain (|)	Inclusion	RG
16. Psychiatric disorders	+	-	Uncertain (|)	Inclusion	RG
17. Diabetes	-	+	Uncertain (|)	Inclusion	RG
18. Pulmonary diseases	+	+	Agreement	Inclusion	SE
19. Cardiovascular diseases	-	+	Uncertain (|)	Inclusion	RG
20. Hypertension	-	+	Uncertain (|)	Inclusion	RG
21. Parkinson’s disease	+	-	Uncertain (|)	Inclusion	RG
22. Sleep apnoea	-	-	Uncertain (&)	Inclusion	RG
23. End-stage renal disease	+	+	Agreement	Inclusion	RG
24. The total number of comorbidities	+	+	Agreement	Exclusion	PD
Medication and addiction related factors					
25. Polypharmacy	+	+	Agreement	Inclusion	SE
26. Benzodiazepines	+	+	Agreement	Inclusion	SE
27. Anticholinergics	+	+	Agreement	Inclusion	SE
28. Problematic alcohol consumption	+	-	Uncertain (|)	Inclusion	RG
29. Tobacco/nicotine dependence	-	-	Uncertain (&)	Inclusion	RG
Intraoperative factors					
30. Blood loss	+	+	Agreement	Inclusion	SE
31. Unplanned prolonged surgery duration	+	+	Agreement	Inclusion	SE
32. Further complications (to be filled out)	+	-	Uncertain (|)	Inclusion	RG
Postoperative factors					
33. Urinary catheterization	+	+	Agreement	Inclusion	SE
34. Physical restraint use	+	+	Agreement	Inclusion	SE
35. Pain	+	+	Agreement	Inclusion	SE
36. Sleep disturbances	+	+	Agreement	Inclusion	SE
37. Infection	+	+	Agreement	Inclusion	SE
38. Hypoxia	+	+	Agreement	Inclusion	SE
Factors derived from qualitative interviews					
39. Type of surgical procedure	+	+	Agreement	Exclusion	PD
40. Invasiveness of the procedure	+	-	Uncertain (|)	Exclusion	RG
41. Type of anaesthesia procedure	-	-	Uncertain (&)	Exclusion	RG
42. Duration of anaesthesia	-	-	Uncertain (&)	Exclusion	RG
43. Medication list of anticholinergics	-	-	Uncertain (&)	Exclusion	RG
44. Bladder dysfunction	-	-	Uncertain (&)	Exclusion	RG
45. Constipation	-	-	Uncertain (&)	Inclusion	SE

* Agreement measured by RAM classical agreement index (AI, i.e., agreement = less than 5 expert answers outside of median category); | relevance OR feasibility uncertain; & relevance AND feasibility uncertain; SE = Decision based on scientific evidence (SE); RG = Decision reached by discussion within research group (RG); PD = Decision due to redundancy of this information already included in the patient data section of the GeDePa.

## Data Availability

The data supporting the conclusions of this article will be made available by the authors on request.

## Supplementary Materials

The following supporting information can be downloaded at: https://www.mdpi.com/article/10.3390/geriatrics11010010/s1, S1: Included Literature and Risk Factors; S2: Interview Guide; S3: Final GeDePa Version [7,9,13,14,35,50,51,52,53,54,55,56,57,58,59,60,61,62,63,64,65].

## Abbreviations

The following abbreviations are used in this manuscript:

A4	DIN A4 Paper Sheet Format
AI	Agreement Index
APC	Article Processing Charges
ASA	American Society of Anesthesiologists Risk Classification
AT	4AT Screening Tool for Delirium
CAT	Category (Median category)
CDK	Christian-Doppler-Clinic
CUP	Codice Unico di Progetto (Italian Fiscal Project Code)
DI	Disagreement Index
DOS	DOS Delirium Observation Screening Tool for Delirium
DSM-5	Diagnostic and Statistical Manual of Mental Disorders Fifth Edition
GeDePa	Geriatric Delirium Pass
GP/GPs	General Practitioner/s
HCP/HCPs	Healthcare Professional/s
HELP	Hospital Elder Life Program
IBM	IBM SPSS v29
IPR	Interpercentile Range
IPRAS	IPR Adjusted for Asymmetry
M	Mean (arithmetic)
MAXQDA	MAXQDA (Qualitative Analysis Software)
Md	Median
PIPRA	Pre-Interventional Preventive Risk Assessment
PD	Patient Data
PMU	Paracelsus Medical University
POD	Postoperative Delirium
PROP	Preoperative Examination
R	R Statistical Software
RAM	RAND/UCLA Appropriateness Method
RG	Research Group
SABES	South Tyrolean Health Care Services (Südtiroler Sanitätsbetrieb)
SALK	University Hospital Salzburg
SFPR	South Tyrolean Fund for the Promotion of Scientific Research
SPSS	IBM SPSS (Quantitative Analysis Software)
