# Supplementary material for: Relevance and Feasibility of a “Geriatric Delirium Pass” for Older Patients with Elective Surgeries: Findings from a Multi-Methods Study
Source: Geriatrics (Basel). 2026 Jan 13;11(1):10. doi: 10.3390/geriatrics11010010 (PMC12821389; doi:10.3390/geriatrics11010010)
Supplement: Supplementary file 1 [file geriatrics-11-00010-s001.zip › SupplementaryMaterial_S1.pdf]

Group	Risk factor	Aldecoa, 2017 [1]	Bramley, 2021 [2]	Deeken, 2022 [3]	DGG_S3, 2022 [4]	Gracie, 2021 [5]	Hoogma, 2023 [6]	Hughes, 2020 [7]	Mevorach, 2023 [8]	Mossie, 2022 [9]	NICE, 2023 [10]	Ormseth, 2023 [11]	Qi, 2022 [12]	Rong, 2021 [13]	SIGN, 2019 [14]	Wilson, 2020 [15]	Wu, 2020 [16]	Yang, 2020 [17]	Yang, 2021 [18]	Zhao, 2022 [19]	Zhuang, 2022 [20]	Zhou, 2021 [21]	Research Group: *Inclusion into GeDePa*
**Demographics**	Age (advanced)																						YES
	Sex (male)																						YES
	Sex (female)																						YES
	Sex (both sexes)																						NO
	Education (years)																						NO
	Nursing home residency/living in institution																						YES
**Physical/ functional status**	ASA (higher, increasing class)																						YES
	Frailty																						YES
	Functional dependency																						YES
	Sensory impairment (esp.: vision, hearing)																						YES
	Impairment in ADLs																						NO
	Pre-existing immobility / limited mobility																						YES
	Severity of Illness / critical illness (ICU)																						NO
	Malnutrition																						NO
	Body Mass Index (lower)																						NO
	Body Mass Index (high-obesity/low-underweight)																						NO
**Comorbidity**	Comorbidities (multiple present) / scores																						YES
Neurological comorbidities	Cognitive dysfunctions/impairment/function																						YES
	Dementia																						YES
	Psychiatric disorders, mental illness, depression																						YES
	Neurological disorders (unspecific)																						NO
	Cerebrovascular disease																						NO
	Prior stroke																						YES
	History of delirium																						YES
Cardiac comorbidities in general (without specific comorbidity)	Cardiovascular disease /events																						NO
	Heart failure, structural disease																						YES
	Ischemic heart disease																						YES
Further comorbidities	Diabetes Mellitus																						YES
	End stage renal failure																						NO
	COPD or OSA																						YES
	Parkinson's disease																						YES
	Obstructive sleep apnoea																						YES

	Sleep disorders																						NO
	Hypertension																						NO
**Health status-related conditions**	Chronic pain, pain (preoperative)																						YES
	Infection/intoxication																						NO
	Hypoxia																						NO
	Disorientation																						NO
	Preoperative fluid fasting / dehydration																						YES
	Urinary retention																						NO
	Constipation																						YES
**Drugs**	Polypharmacy / multiple medicines																						YES
Medications (preoperative)	Drugs with anticholinergic effects																						NO
	Benzodiazepines																						YES
	Diphenhydramine																						NO
	Scopolamine																						NO
	Ketamine																						NO
	Meperidine																						NO
	Morphine																						YES
	Zolpidem																						NO
	Histamine-receptor antagonists																						NO
	Psychotic drugs																						NO
	ACEI use																						NO
	Beta-blocker use																						NO
	Sedative-hypnotics																						NO
Drug misuse/consumption	Alcohol-related disorders, alcohol or drug abuse																						YES
	Smoking/tobacco use																						YES
**Laboratory/ Biomarker**	Albumin (low)																						NO
	Interleukin-6 serum																						YES
	CRP																						YES
	Electrolyte disturbances																						NO
	Total protein																						NO
	Haemoglobin																						NO
	Hyponatraemia or hypernatremia																						NO
	Postoperative sodium																						NO
	(Preoperative) Anaemia																						NO
**Perioperative factors**																							
Surgery-related	Site of surgery																						YES
	Type of surgery																						YES

	Cardiac surgery																					YES
	Hip surgery / current hip fracture																					YES
	Knee replacement (compared to hip)																					YES
	Cardiopulmonary bypass																					NO
	Complexity of surgery																					NO
	Duration of surgery																					YES
	Delay of surgery																					NO
Events/conditions during surgery	Bleeding while surgery/blood loss																					YES
	Intra-operative blood transfusion																					YES
	intra-operative fluid administration																					NO
	Anticholinergic medication																					NO
	Use of Benzodiazepines																					NO
	Mechanical ventilation/intubation																					NO
	Glycaemic control																					NO
	Blood pressure																					NO
	Pressure abnormalities (hypotension/hypertension)																					NO
	Hyperoxia																					YES
	Fixation / physical restraint																					NO
Anaesthesia	Anaesthesia type, length and depth																					NO
	Spinal anaesthesia (compared to general)																					YES
	Regional anaesthesia																					NO
	General anaesthesia (general risk factor)																					NO
**Postoperative factors**	Postoperative pain																					YES
	(postoperative) Anaemia																					NO
	Sleep disturbances/deprivation																					YES
	Renal insufficiency																					NO
	Atrial fibrillation																					NO
	Infection																					NO
	Hypoxemia																					NO
	Mechanical ventilation																					NO
	Length of Stay / longer hospitalization																					NO

Legend (suggestive evidence per risk factor)

no hint or ≤5 primary studies

6-≤20 primary studies

>20 primary studies

References (18 systematic reviews, 3 guidelines) used in GeDePa-phase 1 to identify relevant risk factors for postoperative delirium

1. Aldecoa, C.; Bettelli, G.; Bilotta, F.; Sanders, R.D.; Audisio, R.; Borozdina, A.; Cherubini, A.; Jones, C.; Kehlet, H.; MacLullich, A.; et al. European Society of Anaesthesiology evidence-based and consensus-based guideline on postoperative delirium. *Eur J Anaesthesiol* **2017**, *34*, 192-214, doi:10.1097/EJA.0000000000000594.
2. Bramley, P.; McArthur, K.; Blayney, A.; McCullagh, I. Risk factors for postoperative delirium: An umbrella review of systematic reviews. *Int J Surg* **2021**, *93*, 106063, doi:10.1016/j.ijsu.2021.106063.
3. Deeken, F.; Sanchez, A.; Rapp, M.A.; Denkinger, M.; Brefka, S.; Spank, J.; Bruns, C.; von Arnim, C.A.F.; Kuster, O.C.; Conzelmann, L.O.; et al. Outcomes of a Delirium Prevention Program in Older Persons After Elective Surgery: A Stepped-Wedge Cluster Randomized Clinical Trial. *JAMA Surg* **2022**, *157*, e216370, doi:10.1001/jamasurg.2021.6370.
4. Deutsche Gesellschaft für Geriatrie (DGG). S1-Leitlinie Geriatrisches Assessment der Stufe 2 - Living Guideline. **2022**.
5. Gracie, T.J.; Caufield-Noll, C.; Wang, N.Y.; Sieber, F.E. The Association of Preoperative Frailty and Postoperative Delirium: A Meta-analysis. *Anesth Analg* **2021**, *133*, 314-323, doi:10.1213/ANE.0000000000005609.
6. Hoogma, D.; Milisen, K.; Rex, S.; Al tmimi, L. Postoperative delirium: identifying the patient at risk and altering the course: A narrative review. *European Journal of Anaesthesiology and Intensive Care* **2023**, *2*, doi:e0022.
7. Hughes, C.G.; Boncyk, C.S.; Culley, D.J.; Fleisher, L.A.; Leung, J.M.; McDonagh, D.L.; Gan, T.J.; McEvoy, M.D.; Miller, T.E.; Perioperative Quality Initiative, W. American Society for Enhanced Recovery and Perioperative Quality Initiative Joint Consensus Statement on Postoperative Delirium Prevention. *Anesth Analg* **2020**, *130*, 1572-1590, doi:10.1213/ANE.0000000000004641.
8. Mevorach, L.; Forookhi, A.; Farcomeni, A.; Romagnoli, S.; Bilotta, F. Perioperative risk factors associated with increased incidence of postoperative delirium: systematic review, meta-analysis, and Grading of Recommendations Assessment, Development, and Evaluation system report of clinical literature. *Br J Anaesth* **2023**, *130*, e254-e262, doi:10.1016/j.bja.2022.05.032.
9. Mossie, A.; Regasa, T.; Neme, D.; Awoke, Z.; Zemedkun, A.; Hailu, S. Evidence-Based Guideline on Management of Postoperative Delirium in Older People for Low Resource Setting: Systematic Review Article. *Int J Gen Med* **2022**, *15*, 4053-4065, doi:10.2147/IJGM.S349232.
10. National Institute for Health and Care Excellence (NICE). Delirium: prevention, diagnosis and management in hospital and long-term care. **2023**.
11. Ormseth, C.H.; LaHue, S.C.; Oldham, M.A.; Josephson, S.A.; Whitaker, E.; Douglas, V.C. Predisposing and Precipitating Factors Associated With Delirium: A Systematic Review. *JAMA Netw Open* **2023**, *6*, e2249950, doi:10.1001/jamanetworkopen.2022.49950.
12. Qi, Y.M.; Li, Y.J.; Zou, J.H.; Qiu, X.D.; Sun, J.; Rui, Y.F. Risk factors for postoperative delirium in geriatric patients with hip fracture: A systematic review and meta-analysis. *Front Aging Neurosci* **2022**, *14*, 960364, doi:10.3389/fnagi.2022.960364.
13. Rong, X.; Ding, Z.C.; Yu, H.D.; Yao, S.Y.; Zhou, Z.K. Risk factors of postoperative delirium in the knee and hip replacement patients: a systematic review and meta-analysis. *J Orthop Surg Res* **2021**, *16*, 76, doi:10.1186/s13018-020-02127-1.
14. Scottish Intercollegiate Guidelines Network (SIGN). Risk reduction and management of delirium. **2019**.
15. Wilson, J.E.; Mart, M.F.; Cunningham, C.; Shehabi, Y.; Girard, T.D.; MacLullich, A.M.J.; Slooter, A.J.C.; Ely, E.W. Delirium. *Nat Rev Dis Primers* **2020**, *6*, 90, doi:10.1038/s41572-020-00223-4.
16. Wu, J.; Yin, Y.; Jin, M.; Li, B. The risk factors for postoperative delirium in adult patients after hip fracture surgery: a systematic review and meta-analysis. *Int J Geriatr Psychiatry* **2021**, *36*, 3-14, doi:10.1002/gps.5408.
17. Yang, Z.; Wang, X.F.; Yang, L.F.; Fang, C.; Gu, X.K.; Guo, H.W. Prevalence and risk factors for postoperative delirium in patients with colorectal carcinoma: a systematic review and meta-analysis. *Int J Colorectal Dis* **2020**, *35*, 547-557, doi:10.1007/s00384-020-03505-1.
18. Yang, Y.; Zhao, X.; Gao, L.; Wang, Y.; Wang, J. Incidence and associated factors of delirium after orthopedic surgery in elderly patients: a systematic review and meta-analysis. *Aging Clin Exp Res* **2021**, *33*, 1493-1506, doi:10.1007/s40520-020-01674-1.
19. Zhao, J.; Liang, G.; Hong, K.; Pan, J.; Luo, M.; Liu, J.; Huang, B. Risk factors for postoperative delirium following total hip or knee arthroplasty: A meta-analysis. *Front Psychol* **2022**, *13*, 993136, doi:10.3389/fpsyg.2022.993136.
20. Zhuang, X.; He, Y.; Liu, Y.; Li, J.; Ma, W. The effects of anesthesia methods and anesthetics on postoperative delirium in the elderly patients: A systematic review and network meta-analysis. *Front Aging Neurosci* **2022**, *14*, 935716, doi:10.3389/fnagi.2022.935716.
21. Zhou, Q.; Zhou, X.; Zhang, Y.; Hou, M.; Tian, X.; Yang, H.; He, F.; Chen, X.; Liu, T. Predictors of postoperative delirium in elderly patients following total hip and knee arthroplasty: a systematic review and meta-analysis. *BMC Musculoskelet Disord* **2021**, *22*, 945, doi:10.1186/s12891-021-04825-1.
