# Supplementary material for: Relevance and Feasibility of a “Geriatric Delirium Pass” for Older Patients with Elective Surgeries: Findings from a Multi-Methods Study
Source: Geriatrics (Basel). 2026 Jan 13;11(1):10. doi: 10.3390/geriatrics11010010 (PMC12821389; doi:10.3390/geriatrics11010010)
Supplement: Supplementary file 1 [file geriatrics-11-00010-s001.zip › SupplementaryMaterial_S2.pdf]

GeDePa-Study: Interview Guide – Expert Interviews

(English version for publication purpose)

Optimization of interprofessional perioperative care of geriatric patients at risk of delirium after elective surgery – A multi-method study on the development and evaluation of the feasibility and relevance of a Geriatric Delirium Pass (GeDePa)

Procedure (information for the interviewer)

Joining Phase

- Welcome and introduction of interviewer
- Creating a comfortable atmosphere
- Information and clarification about the study
- Voluntary participation
- Data protection and explanation of digital recording
- Review of informed consent
- Explanation of interview procedure
- Completion of short demographic/professional questionnaire
- Start recording
- Do not read the questions like standardizes survey questions but stick to the content.
- Begin with Block 1 (introductory/icebreaker), proceed flexibly with follow-up questions, and end with closing question

Off-the-record Phase

- Notify participants that recording has ended
- Short reflection on the interview with option for feedback
- Information about next project steps (transcription, pseudonymization, online survey participation)
- Clarify whether follow-up contact is possible for questions
- Provide project team contact information

Geriatric Delirium Pass*

Inform – Raise Awareness – Document – Alert – Prevent

Pseudonomized Code: ________________________________

Demographic/Professional Background – Short Questionnaire *[documented by the interviewer]*

a. Gender ☐ female ☐ male

b. Profession ________________________________

c. Discipline (HCP) ________________________________

d. Relevant additional qualifications ________________________________

____

____

____

e. Total years of professional experience _________ years

f. Years in current position _________ years

g. Work setting ☐ (rather) rural ☐ (rather) urban

**Thematic block 1: Frequency and Relevance of Delirium *[Introductory]***	
**Main questions**	**Themes for probing questions**
▪ How relevant is delirium in your daily professional routine with older patients?<br>▪ How is delirium considered in your routines with older patients undergoing elective surgery?<br>▪ What are the main challenges in your *[setting]* regarding POD?<br>▪ *Optional*: Where do you see potential for optimization in your area?	*Frequency and relevance in everyday care*<br>*Typical measures and procedures*<br>*Delirium as a priority in the current care of older patients undergoing elective surgery*<br>*Basic aspects with potential for optimization in care*
**Thematic block 2: Early Risk Detection and Care Processes**	
**Main questions**	**Themes for probing questions**
When you think in general about the intersectoral continuum of care for older patients undergoing elective surgery (e.g., a 70-year-old woman receiving a hip replacement):<br>▪ In your setting, how is delirium risk assessed for older patients undergoing elective surgery?<br>▪ What preventive measures are undertaken? Which regional structures and processes exist?<br>▪ At which points of care (e.g., GP, specialist, anesthesiology, nursing) are early detection measures currently applied?<br>▪ How does information exchange between sectors, settings, and professions take place?<br>▪ Which health professions are central to POD prevention across the continuum of care?<br>▪ Follow-up: Which challenges and opportunities for optimization do you see?<br>▪ What would an ideal process for POD prevention look like?	*Concrete measures applied: identification, assessments, screenings, key risk factors, geriatric assessment*<br>*Interprofessional collaboration, interfaces, communication, information flow*<br>*Information from and support for relatives*
**Thematic block 3: Practicability of the Delirium Pass**<br>*[Instruction: Repeat the core idea of the GeDePa and present, i.e., show and hand over the draft version as example.]*	
**Main questions**	**Themes for probing questions**
▪ What is your first impression of the GeDePa in terms of format and appearance?<br>▪ What are your spontaneous thoughts about using such a pass in your setting?<br>▪ How do you assess its practicability in everyday work-	*User-friendliness*<br>*Scope, time required for completion, format and layout*<br>*Applicability, willingness to use in the setting*

flows?<br>▪ What specific benefits could it provide in your area?<br>▪ What benefits could other settings/professions gain from the GeDePa?	*Suggestions and tips for design/improvement*<br>*Feasibility, practical implementation, effort*<br>*Meaningfulness, potential, benefit*
**Thematic block 4: Content and Risk Factors**	
**Main questions**	**Themes for probing questions**
▪ What is your first impression of the GeDePa content?<br>▪ Which information and components should be mandatory? Which would be supplementary?<br>▪ Which information could be collected at which point of care?<br>▪ Which information could you collect yourself, and which would you need from others?	*Patient-related information and characteristics*<br>*Risk factors in the Delirium Pass: patient-related, environment-related, medication-related, medical-nursing/procedure-related*<br>*Scales and assessments, meaningfulness of ad hoc evaluation of a risk profile*<br>*Preference for detailed risk factor documentation versus short assessment scales for delirium risk*<br>*Own area of competence and responsibility*
Thematic block 5: Facilitators and Barriers *[Closing]*	
**Main questions**	**Themes for probing questions**
Finally, when you as a *[profession]* think about the introduction of a Geriatric Delirium Pass for older patients undergoing elective surgery, aimed at preventing postoperative delirium across the intersectoral continuum of care:<br>▪ What challenges might occur when introducing and applying the GeDePa in your setting?<br>▪ What challenges would you expect in the overall care process?<br>▪ How could its introduction and use be supported?<br>**Closing question**<br>▪ Do you have further comments or improvement suggestions?	*Processes and structures*<br>*Billing, service provision*<br>*Professional groups, interfaces, patient pathways*<br>*Resources, bureaucracy*<br>*Analog versus digital Delirium Pass*<br>*Willingness of patients*

[Setting]: Point-of-Care: primary care, in-hospital geriatric/surgical department

[Professions]: General practitioner, geriatrician, registered nurse, anesthetist
