# Supplementary material for: Relevance and Feasibility of a “Geriatric Delirium Pass” for Older Patients with Elective Surgeries: Findings from a Multi-Methods Study
Source: Geriatrics (Basel). 2026 Jan 13;11(1):10. doi: 10.3390/geriatrics11010010 (PMC12821389; doi:10.3390/geriatrics11010010)
Supplement: Supplementary file 1 [file geriatrics-11-00010-s001.zip › SupplementaryMaterial_S3.pdf]

Geriatric Delirium Pass*

Inform – Raise Awareness – Document – Alert – Prevent

Patient

Surname, Given Name ______________________

Date of Birth ______________________

Age >70 years o ≥ 70 years o < 70 years

Sex (m, w, d) o female o male o divers

Living/Care Situation
(e.g., At home, Nursing/Care Home, Outpatient Care, Informal Caregiving) ______________________

Contact Person/Caregiver ______________________

Referring General Practitioner *(GP)*, Outpatient Clinic (OC), Specialist (SP) ______________________

Diagnoses and Comorbidities

Primary Diagnosis ______________________

Comorbidities *[please check off (x)]* o >2 Comorbidities

o Dementia o Psychiatric Disorder o Hypertension
o Cardiovascular Diseases o Diabetes o Parkinson's
o End-stage Renal Failure o COPD o Sleep Apnoea

Planned Surgical Procedure/Type; Date

____

Preoperative Risk Factors for Postoperative Delirium (Indication of At-Risk Patients)

	GP, Outpatient Clinic, Specialist		Inpatient Care (Anaesthesia, Geriatrics, Orthopedics, Surgery)			
			Nursing		Medical	
Please check off (x) or fill in:	*yes*	*no*	*yes*	*no*	*yes*	*no*
**General Status**						
Sensory Impairment *(vison and/or hearing)*	o	o	o	o	o	o
Frailty[1]	o	o	o	o	o	o
Limited Mobility[2]	o	o	o	o	o	o
Reduced Sleep Quality (e.g., falling/staying asleep, reversed day-night rythm)	o	o	o	o	o	o
Indication of Malnutrition (e.g., BMI < 18)	o	o	o	o	o	o
Laboratory parameters outside normal range *[if yes, please check off]* *o Albumin, o CRP, o Potassium, o Sodium, o Total Protein, o Haemoglobin*	o	o	o	o	o	o
ASA-risk classification > *2*[3]	o	o	o	o	o	o
Delirium or Confusion in the past	o	o	o	o	o	o
Pain (at rest or during activity)[4]	o	o	o	o	o	o
Cognitive Impairment[5]	o	o	o	o	o	o
**Medications, Addictive Behavior**						
Polypharmacy (≥5 prescribed systemic drugs)	o	o	o	o	o	o
Use of benzodiazepines	o	o	o	o	o	o
Medications with anticholinergic effects[6]	o	o	o	o	o	o
Problematic alcohol consumption[7])	o	o	o	o	o	o
Tobacco/Nicotine dependence[7]	o	o	o	o	o	o

Free comment section: Clinical observations and other important notes

Original GeDePa (for Peer Review)

** Developed within the research project "GeDePa – Geriatric Delirium Pass" (2023–2025)*

Turn page…

claudiana – Universitäres Ausbildungszentrum für Gesundheitsberufe / Polo Universitario delle Professioni Sanitarie
PARACELSUS MEDIZINISCHE UNIVERSITÄT
Autonome Provinz Bozen – Südtirol / Provincia Autonoma di Bolzano – Alto Adige / Provinzia Autonoma de Bulsan – Sudtirol
Südtiroler Sanitätsbetrieb / Azienda Sanitaria dell'Alto Adige / Azienda Sanitera de Sudtirol
UNIKLINIKUM SALZBURG

Geriatric Delirium Pass*

Inform – Raise Awareness – Document – Alert – Prevent

Peri- and Postoperative Risk Factors and Events

	Inpatient Care (Anesthesia, Geriatrics, Orthopedics, Surgery)			
	Nursing		Medical	
Please check off (x) or fill in:	*yes*	*no*	*yes*	*no*
**Intraoperative Complications**				
Blood loss, intraoperative transfusion	o	o	o	o
Unplanned prolonged surgery duration	o	o	o	o

… other complications (e.g., intraoperative hypotension, depth of anaesthesia, anaesthesiological events) *[please specify]*:

____

____

**Postoperative Factors**	Nursing yes	Nursing no	Medical yes	Medical no
Urinary retention / catheter	o	o	o	o
Other necessary access (e.g., cannula, central venous catheter, midline)	o	o	o	o
Constipation, abdominal pain, feeling of fullness	o	o	o	o
Need for restraints (e.g., belts, bed rails)	o	o	o	o
Postoperative Pain (at rest or during activity)[4]	o	o	o	o
Indication of infection requiring treatment	o	o	o	o
Hypoxia requiring treatment	o	o	o	o

Documentation of Delirium in the Postoperative Course

Date																																				
Post-OP Day	0 (day of OP)						1						2						3						4						5					
Time-of-day	4	8	12	16	20	24	4	8	12	16	20	24	4	8	12	16	20	24	4	8	12	16	20	24	4	8	12	16	20	24	4	8	12	16	20	24
Signs of a delirium event (using instruments like 4AT, DOS) [X] no, [X] yes																																				
Medical diagnosis [X] yes																																				
Medication upon medical diagnosis? [X] no, [X] yes																																				

Note: *Please enter the date and every 4 hours check "yes" or "no" to indicate whether there are signs of a delirium event, a medical diagnosis, and medication administration for delirium.*

Notes and Recommendations for Identifying Selected Risk Factors

Risk factor or item	Existing diagnoses, assessments | Expert evaluation
[1] Frailty	e.g.: Clinical Frailty Scale (CFS), FRAIL, PRISMA 7, Groningen Frailty Indicator (GFI)
[2] Limited mobility	e.g., refer to Barthel Index items: *Transfer (chair ↔ bed: not possible/only with support vs. independent)* *Mobility/walking (not possible/only with support vs. independent)*
[3] ASA-risk classification	ASA 1-*Healthy patient* | 2-*Patient with mild systemic disease without functional limitations* | 3-*Patient with severe systemic disease with marked limitation* | 4-*Patient with severe systemic disease that is a constant threat to life* | 5-*Moribund patient not to survive without the operation* | 6-*Brain-dead patient whose organs are being removed for donor*
[4] Current Pain	e.g., Numeric Rating Scale (NRS) or Verbal Rating Scale (VRS) at rest and/or during activity, PAINAD (for people with advanced dementia)
[5] cognitive impairment	e.g., MMSE, Mini-Cog, Six-Item Screener (SIS), Six-item Cognitive Impairment Test (6CIT)
[6] Anticholinergic medications	e.g., Tricyclic antidepressants (*amitriptyline, doxepin, imipramine*), SSRIs (*paroxetine*), First-generation antihistamines (*diphenhydramine, dimenhydrinate, hydroxyzine, promethazine*), Antipsychotics (*clozapine, olanzapine, chlorpromazine*), Urological anticholinergics (*oxybutynin, tolterodine, solifenacin*), Antiparkinson drugs (*biperiden, trihexyphenidyl*), Antiemetics (*scopolamine*), Others (*atropine, baclofen, tizanidine*)
[7] Alcohol, tobacco/nicotine use	Alcohol (*♀>2 standard glasses/day, ♂>4 standard glasses/day*), tobacco-/nicotine (*>10 cigarettes/Tag*)

S3 Final GeDePa (Peer Review)
